# High prevalence of subjective cognitive decline in older Chinese adults: a systematic review and meta-analysis

**DOI:** 10.3389/fpubh.2023.1277995

**Published:** 2023-12-01

**Authors:** Chao Xue, Juan Li, Mingqing Hao, Lihua Chen, Zuoxiu Chen, Zeli Tang, Huan Tang, Qian Fang

**Affiliations:** ^1^School of Nursing, Guizhou University of Traditional Chinese Medicine, Guiyang, Guizhou, China; ^2^Department of Nursing, Guizhou Provincial People's Hospital, Guiyang, Guizhou, China; ^3^School of Nursing, Zunyi Medical University, Zunyi, Guizhou, China

**Keywords:** Chinese, subjective cognitive decline, prevalence, meta-analysis, Alzheimer’s disease

## Abstract

**Background:**

Subjective cognitive decline (SCD) is considered a preclinical stage of Alzheimer’s disease. However, reliable prevalence estimates of SCD in the Chinese population are lacking, underscoring the importance of such metrics for policymakers to formulate appropriate healthcare strategies.

**Objective:**

To systematically evaluate SCD prevalence among older Chinese adults.

**Methods:**

PubMed, Web of Science, The Cochrane Library, Embase, CNKI, Wanfang, VIP, CBM, and Airiti Library databases were searched for studies on SCD in older Chinese individuals published before May 2023. Two investigators independently screened the literature, extracted the information, and assessed the bias risk of the included studies. A meta-analysis was then conducted using Stata 16.0 software via a random-effects model to analyze SCD prevalence in older Chinese adults.

**Results:**

A total of 17 studies were included (n = 31,782). The SCD prevalence in older Chinese adults was 46.4% (95% CI, 40.6–52.2%). Further, subgroup analyzes indicated that SCD prevalence was 50.8% in men and 58.9% among women. Additionally, SCD prevalence in individuals aged 60–69, 70–79, and ≥ 80 years was 38.0, 45.2, and 60.3%, respectively. Furthermore, SCD prevalence in older adults with BMI <18.5, 18.5–24.0, and > 24.0 was 59.3, 54.0, and 52.9%, respectively. Geographically, SCD prevalence among older Chinese individuals was 41.3% in North China and 50.0% in South China. In terms of residence, SCD prevalence was 47.1% in urban residents and 50.0% among rural residents. As for retired individuals, SCD prevalence was 44.2% in non-manual workers and 49.2% among manual workers. In the case of education, individuals with an education level of “elementary school and below” had an SCD prevalence rate of 62.8%; “middle school, “52.4%; “high school, “55.0%; and “college and above, “51.3%. Finally, SCD prevalence was lower among married individuals with surviving spouses than in single adults who were divorced, widowed, or unmarried.

**Conclusion:**

Our systematic review and meta-analysis identified significant and widespread SCD prevalence in the older population in China. Therefore, our review findings highlight the urgent requirement for medical institutions and policymakers across all levels to prioritize and rapidly develop and implement comprehensive preventive and therapeutic strategies for SCD.

**Systematic review registration**: https://www.crd.york.ac.uk/prospero/display_record.php?ID=CRD42023406950, identifier: CRD42023406950.

## 1 Introduction

Alzheimer’s disease (AD) is the leading cause of dementia (1, 2), with the latest data from the 2023 Alzheimer’s Disease Facts and Figures report indicating that approximately 10% of the world’s population aged over 65 years is living with AD (1). Moreover, the number of individuals with AD is projected to exceed 131 million by 2050 (3). Furthermore, the current status of AD epidemiology in China appears to be much worse than the global average. China, which has the fastest-growing older population in the world, may account for approximately half of the global AD population by 2050 (4, 5). With the increasing aging population in China, dementia (particularly AD) incidence is escalating rapidly (6, 7). AD is now the fifth leading cause of death in China (4, 8), and it is seriously affecting the physical and mental health of patients and family caregivers, utilizing national public health resources, and exacerbating the social and economic burden (1, 4, 5, 9).

Recent studies have indicated that preventive strategies for AD are the key to reducing this disease prevalence (1). Accurate identification and scientific interventions during the early AD stages can result in effective treatments for its comprehensive management (10–12), thereby reducing the financial and caregiving burdens associated with this neurodegenerative disease. In 2014, Jessen et al. (13) formally proposed the concept of subjective cognitive decline (SCD), referring to an individual’s subjective perception of a persistent decline in cognitive function or memory compared to their previous normal state, irrespective of the normal results on objective neuropsychological examinations (13–15). Additionally, Jessen et al. (13, 16) suggested that SCD might serve as a preclinical stage of AD, aiding in the prediction of AD development. SCD has garnered considerable worldwide attention from scholars since its initial proposal, becoming a focal point in the fields of geriatric neurology and cognition (14, 17, 18). A meta-analysis of longitudinal studies found that SCD is associated with an increased risk of developing dementia (HR = 1.90, 95% CI 1.52–2.36; OR = 2.48, 95% CI 1.97–3.14), with an average progression to dementia of 10% (19). However, considering that SCD-related research in China was initiated relatively later, its prevalence and severity may be underestimated in the older adult population of this country.

Epidemiological studies have been prioritized in the Beijing area since 2015 to understand SCD prevalence among older Chinese adults and guide policy-making to address this trend (20). Although concerns regarding SCD emerging as a major public health issue in China have increased (21–24), epidemiological data on SCD in China’s older population are scarce, with existing surveys being limited to the specific characteristics, study methodologies, identification, and classification of SCD (20, 25, 26). Thus, direct comparisons between individual studies are constrained.

In this systematic review and meta-analysis, our primary objective was to evaluate SCD prevalence in older Chinese adults by analyzing all available published data related to this topic. Additionally, we aimed to compare prevalence estimates among various subgroups, including those based on sex, age, body mass index (BMI), marital status, education level, geographical region, residence, and occupation type before retirement.

## 2 Methods

This review adhered to the guidelines of the Preferred Reporting Items for Systematic Reviews and Meta-Analyzes (PRISMA) (27) and the Meta-Analysis of Observational Studies in Epidemiology (28). Furthermore, this review was registered with PROSPERO (registration no. CRD42023406950).

### 2.1 Search strategy

Here, we systematically searched nine databases, including Embase, PubMed, The Cochrane Library, Web of Science, China National Knowledge Infrastructure Database, Wanfang Database, Chinese Biomedical Literature Database, Chinese Scientific Journal Database (VIP database), and Airiti Library, to locate all relevant publications on SCD epidemiology in China. The search period was from the date of database inception to May 1, 2023, with no language restrictions. Before finalizing the search strategy, we consulted with a librarian knowledgeable about systematic review methodology and the process of refining search terms. We also conducted several preliminary searches to obtain the most comprehensive collection of relevant literature. The search terms entered were as follows: (“subjective cognitive decline” OR “subjective memory decline” OR “subjective memory complain*” OR “subjective cognitive complain*” OR “subjective memory loss”) AND (“incidence” OR “prevalence” OR “occurrence” OR “rate*” OR “epidemiology”) AND (“Chinese” OR “China” OR “Hong Kong” OR “Macao” OR “Taiwan”).

### 2.2 Inclusion and exclusion criteria

The inclusion criteria were as follows: (1) studies with participants older than 60 years, (2) those including participants residing in China, including mainland China, Hong Kong, Macao, and Taiwan, (3) observational research study types, including cross-sectional and cohort studies, (4) those that applied the SCD diagnostic criteria (13, 16) and reported SCD prevalence, and (5) those published in Chinese or English. The exclusion criteria were as follows: (1) non-research articles such as reviews, case reports, comments, letters, and editorials, (2) duplicate publications, (3) studies without full-text availability, or (4) those of low research quality, i.e., Agency for Healthcare Research and Quality (AHRQ) quality assessment scores <4 (29).

### 2.3 Study selection and data extraction

All of the retrieved literature was imported into Endnote 20. After deleting duplicate initial search results within and between different databases, the two authors (C XUE and M HAO) independently reviewed the titles and abstracts to identify potentially eligible articles requiring a full assessment. In the case of multiple publications utilizing the same sample or original data, the publication with the largest sample size and the most thorough information on data extraction was given preference. To resolve differing opinions between the two reviewers, a third researcher (L CHEN) was consulted and asked to deliberate and vote on the specific issue. The two reviewers (C XUE and M HAO) then used a standardized form to independently extract and record the following information: first author, publication year, geographical location, sample size, number and age of participants, assessment tools, and SCD prevalence.

### 2.4 Quality assessment

Two researchers (C XUE and M HAO) independently evaluated the bias risk in the included studies and cross-checked their assessments. In the case of disagreement, a discussion or a third-party consultation was used to obtain a judgment. To examine the methodological quality of the cross-sectional studies, we used an 11-item checklist recommended by the AHRQ (30). In this checklist, each item contained three options: “Yes, ““No, “or “Not clear.” A score of 1 point was given for “Yes,” whereas 0 points were scored for “No” and “Not clear.” The overall score ranged from 0 to 11, wherein articles with scores of 0–3 were classified as low quality; 4–7, moderate quality; and 8–11, high quality.

### 2.5 Statistical analysis

The metaprop command in Stata 16.0 (StataCorp, College Station, TX, United States) was used to statistically analyze SCD prevalence in the older Chinese population. Furthermore, the *χ*^2^ (with a test level of *α* = 0.1) and I^2^ tests were employed to analyze the heterogeneity of the results. For study results with no statistical heterogeneity (*p* ≥ 0.1 and I^2^ ≤ 50%), a fixed-effects model was utilized for the meta-analysis. Conversely, in those involving statistical heterogeneity (*p* < 0.1 or I^2^ > 50%), the results were further analyzed to identify the heterogeneity source (31). After eliminating the obvious clinical heterogeneity, a random-effects model was employed for the meta-analysis. The significance level for the meta-analysis was *α* = 0.05. Additionally, subgroup analysis was performed to evaluate SCD prevalence based on sex, age, BMI, marital status, education level, occupation type before retirement, and geographical region. Additionally, sensitivity analysis was conducted by sequentially excluding each study and then recalculating the pooled estimates of the remaining studies to determine the effect of individual studies on the overall prevalence estimates. Lastly, publication bias was evaluated using funnel plots and Egger’s test (32, 33).

## 3 Results

### 3.1 Study selection and characteristics

**Figure 1:**
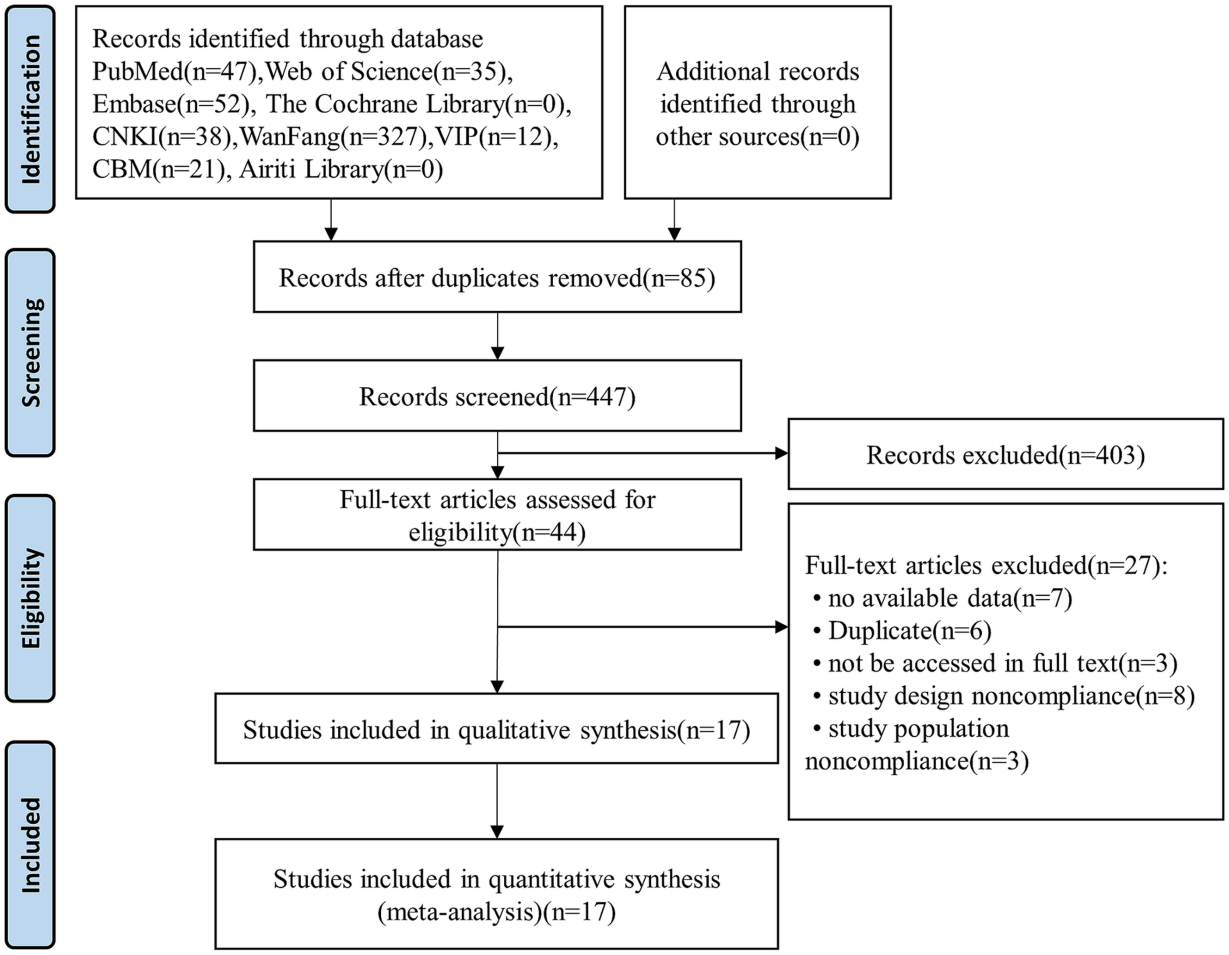
PRISMA flowchart for study selection.

The initial database search yielded 532 relevant literature, which was imported into the EndNote Literature Manager software. After deleting duplicates, 447 studies remained. Next, the two researchers (C XUE and M HAO) independently read the titles and abstracts and deleted studies that did not meet the review’s inclusion and exclusion criteria, resulting in 44 remaining studies. Further, the full text of the articles was read, leading to the deletion of 27 articles. Among them, seven had no available data, six were duplicates of previous publications, three did not have accessible full text, eight consisted of noncompliant study designs, and three involved noncompliant study populations. References of the included literature were also checked to identify any potentially missed publications. After this step-by-step filtering procedure, 17 studies were included in the final meta-analysis. The included studies had a total sample size of 31,782 participants, of which 14,390 were from the SCD population. The literature screening process and results are shown in Figure 1.

Table 1 presents the characteristics of the studies included in the meta-analysis. A total of 17 studies were included, comprising 31,782 older adults. Regarding the inclusion criteria, all 17 studies stated that the study subjects were permanent local residents aged 60 years and above. Additionally, they were required to have normal objective cognitive function and daily living ability, as well as the ability to answer the relevant questionnaires independently. In terms of the exclusion criteria, all 17 studies mentioned excluding mild cognitive impairment and dementia. Thirteen studies mentioned excluding diseases that might affect objective cognitive function. Furthermore, 14 studies mentioned excluding severe mental disorders, 10 studies mentioned excluding severe visual or hearing impairment that could hinder normal assessment, and 9 studies mentioned excluding severe organ failure. Moreover, 6 studies mentioned excluding substance abuse, 2 studies mentioned excluding alcohol dependence, and 2 studies mentioned excluding participation in other interventional studies. Lastly, only 1 study mentioned excluding shift-workers. The sample sizes in these studies ranged from 173 to 10,312 individuals. The data examined in these studies were obtained from the years 2016 to 2023. The studies were conducted in 16 administrative divisions, including provinces, autonomous regions, and municipalities directly under the central government in China. Geographically, 10 studies were conducted in South China, whereas seven in North China. In terms of residence, 12 studies (70.6%) were performed in urban settings, three in rural areas (17.6%), and two (11.8%) involved urban and rural locations. Furthermore, the evaluation of literature quality yielded AHRQ scores of 5–9 for the included articles, indicating medium- to high-quality articles and confirming reliability in our review results.

**Table 1:** Characteristics of the included studies.

References	Research sites	Age	Total sample size	Numbers of SCD	Prevalence of SCD (%)	Assessment tools for SCD	AHRQ score
Song et al. (34)	Guangzhou	≥60	612	250	40.85	SCD-Q9	8
Yu et al. (35)	Baotou	≥60	1,120	491	43.84	SCD-Q9	8
Ai et al. (36)	Shanghai	60–85	173	132	76.3	Self-developed SCD-Q	8
Dong et al. (37)	Suzhou, Anhui Province	≥60	264	153	57.95	SCD-Q9	9
Wen et al. (38)	Northern China	≥65	1,165	493	42.32	Self-developed SCD-Q	7
Hu et al. (39)	Guangzhou	≥60	212	86	40.57	SCD-Q9	9
Huang et al. (40)	Hubei Province	≥65	10,312	5,134	48.93	SCD-Q9	8
Zhou et al. (41)	Beijing	≥60	337	111	32.9	SCD-Q9	8
Yang et al. (42)	Wuhan	≥60	211	105	49.8	SCD-Q	9
Wang et al. (43)	Yanggu county in Shandong Province	≥60	5,765	2,654	46.04	SCD-Q9	9
Chen et al. (44)	Guangzhou	≥60	212	42	19.81	Self-developed SCD-Q	7
Han et al. (45)	Xiamen	≥60	1,318	749	56.8	SCD complaints	9
Ruan et al. (46)	Changning, Shanghai	≥60	5,328	2,217	41.61	SCD-Q	9
Lin et al. (25)	Guangzhou	≥60	688	402	58.4	SCD-Q9	9
Hao et al. (20)	Shunyi, Beijing	60–80	2,689	506	18.8	SCD-Q9	9
Su et al. (26)	Daqing	≥60	308	155	49.68	SCD-Q	9
Xiao et al. (47)	Shanghai	≥60	1,068	710	66.5	COWAT	5

SCD-Q9, Subjective Cognitive Decline-Questionnaire 9; SCD-Q, Subjective Cognitive Decline-Questionnaire; COWAT, Controlled Oral Word Association Test; AHRQ, Agency for Healthcare Research and Quality.

### 3.2 Pooled prevalence of SCD

**Figure 2:**
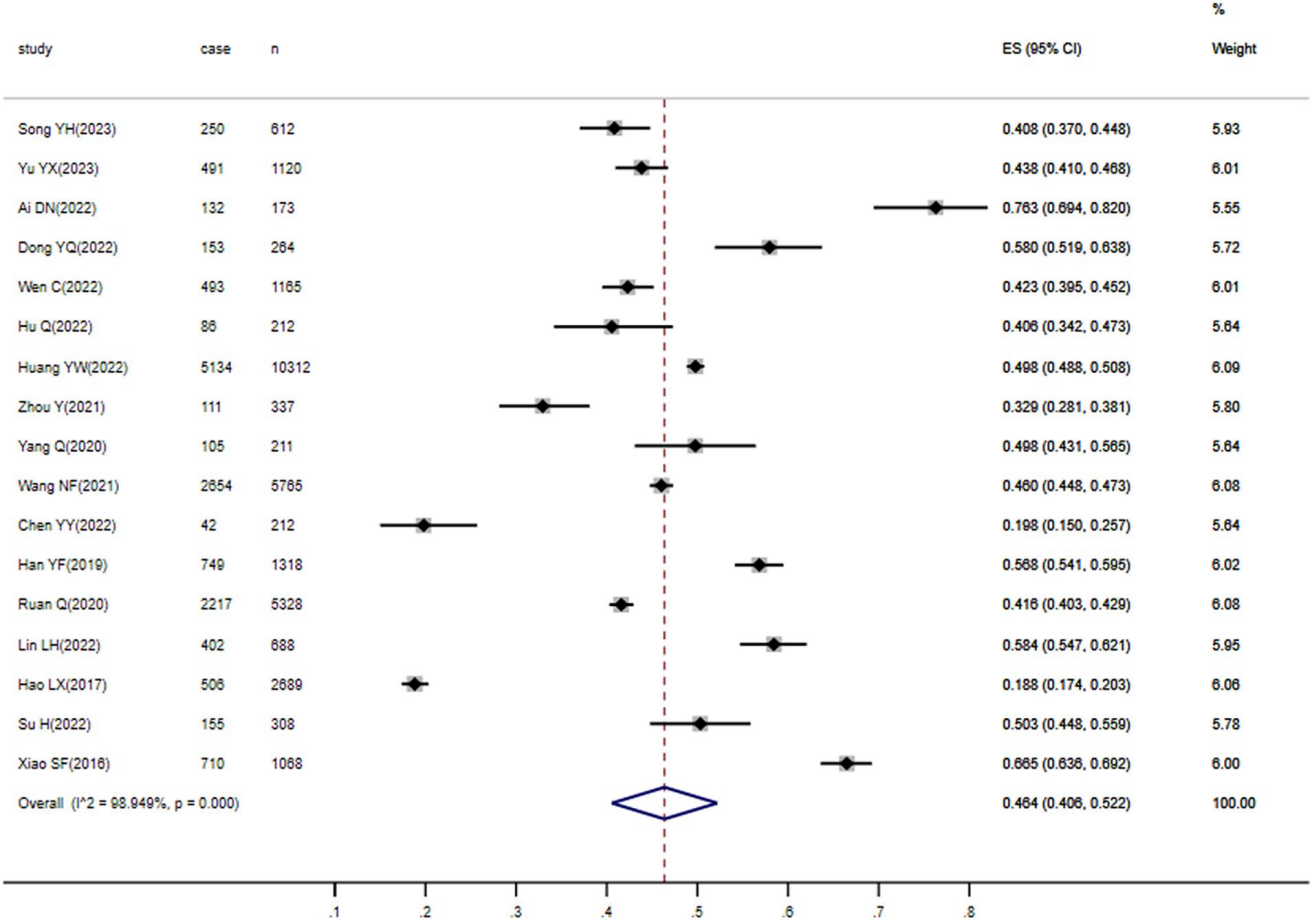
Forest plot of overall SCD prevalence in Chinese older adults.

As illustrated in Figure 2, SCD prevalence in older Chinese individuals ranged from 18.8 to 76.3%. Further, the estimated pooled prevalence of SCD in the 17 studies was 46.4% (95% CI, 40.6–52.2%).

### 3.3 Subgroup analyzes

Table 2 presents the subgroup analysis findings of SCD prevalence among older Chinese adults in the included studies. The results indicated that men had a significantly lower SCD prevalence (50.8, 95% CI, 41.4–60.2%) than women (58.9, 95% CI, 50.3–67.3%, *p* < 0.001).

**Table 2:** Various subgroup analyzes of SCD prevalence in Chinese older adults.

Subtype	Numbers of studies	Heterogeneity assessment		Random/fixed effects model	Meta-analysis results	*p*-value across subgroups
		*I*^2^(%)	*P*		Prevalence of SCD (95%CI)	
*Gender*						
Male	11	98.6%	<0.001	Random	50.8% (41.4, 60.2%)	<0.001
Female	11	98.6%	<0.001	Random	58.9% (50.3, 67.3%)	
*Age, years*						
60–69	8	99.4%	<0.001	Random	38.0% (21.4, 56.2%)	<0.001
70–79	9	98.5%	<0.001	Random	45.2% (34.9, 56.7%)	
≥80	8	93.4%	<0.001	Random	60.3% (50.8, 69.5%)	
*BMI, Kg/m^2^*						
<18.5	5	77.3%	0.001	Random	59.3% (45.6, 72.4%)	0.051
18.5 ~ 24.0	5	96.2%	<0.001	Random	54.0% (43.4, 64.5%)	
>24.0	5	97.5%	<0.001	Random	52.9% (40.1, 65.5%)	
*Education level*						
Primary and Illiteracy	9	95.0%	<0.001	Random	62.8% (56.8, 68.5%)	<0.001
middle school	5	97.5%	<0.001	Random	52.4% (43.3, 61.5%)	
high school	4	93.4%	<0.001	Random	55.0% (39.2, 70.2%)	
college and above	6	93.4%	<0.001	Random	51.3% (37.7, 64.8%)	
*Region*						
Northern China	7	99.2%	<0.001	Random	41.3% (30.4, 52.6%)	<0.001
Southern China	10	98.1%	<0.001	Random	50.0% (44.1, 56.0%)	
*Residence*						
City	14	99.1%	<0.001	Random	47.1% (38.3, 56.0%)	0.003
Rural	5	99.6%	<0.001	Random	50.0% (30.3, 69.8%)	
*Married status*						
surviving spouse	7	99.4%	<0.001	Random	51.6% (38.8, 64.2%)	0.38
no spouse	7	92.0%	<0.001	Random	58.2% (50.3, 65.8%)	
*Occupation before*						
Manual labor	3	98.7%	<0.001	Random	49.2% (27.0, 71.7%)	<0.001
Mental labor	3	93.4%	<0.001	Random	44.2% (20.2, 69.8%)	

Additionally, SCD prevalence was found to significantly increase with age (*p* < 0.001), with the 60–69 years age group exhibiting an SCD prevalence of 38.0% (95% CI, 21.4–56.2%); 70–79 years, 45.2% (95% CI, 34.9–56.7%); and ≥ 80 years, 60.3% (95% CI, 50.8–69.5%).

Furthermore, SCD prevalence in older adults decreased with increasing BMI. In particular, SCD prevalence in older adults with BMI <18.5, 18.5–24.0, and > 24.0 was 59.3% (95% CI, 45.6–72.4%), 54.0% (95% CI, 43.4–64.5%), and 52.9% (95% CI, 40.1–65.5%), respectively. Although this difference was only marginally significant (*p* = 0.051), this observation is still worth noting.

In terms of geographical regions, SCD prevalence significantly varied (*p* < 0.001) between older adults in South China (50.0, 95% CI, 44.1–56.0%) and those from North China (41.3, 95% CI, 30.4–52.6%). Furthermore, individuals residing in rural areas exhibited significantly higher SCD prevalence (50.0, 95% CI, 30.3–69.8%) than those in urban locations (47.1, 95% CI, 38.3–56.0%, *p* = 0.003).

Moreover, occupation type before retirement and education level also demonstrated significant differences in SCD prevalence. SCD prevalence among retired non-manual (44.2, 95% CI, 20.2–69.8%) was significantly lower than that in retired manual workers (49.2, 95% CI, 27.0–71.7%, *p* < 0.001). As for education level, SCD prevalence decreased as education level increased, with a significant difference among the four education levels (*p* < 0.001), including “elementary school and below,” “middle school,” “high school,” and “college and above.” Of which, individuals with a higher education level (i.e., college and above) had the lowest prevalence (51.3, 95% CI, 37.7–64.8%), followed by those with secondary education (i.e., middle school [52.4, 95% CI, 43.3–61.5%] and high school [55.0, 95% CI, 39.2–70.2%]). Older adults with education levels of elementary school and below had the highest SCD prevalence of 62.8% (95% CI, 56.8–68.5%).

Lastly, SCD prevalence in married individuals with surviving spouses was lower (51.6%; 95% CI, 38.8–64.2%) than in single adults who were divorced, widowed, or unmarried (58.2, 95% CI, 50.3–65.8%). However, this difference was not statistically significant (*p* = 0.38).

### 3.4 Sensitivity analysis

**Figure 3:**
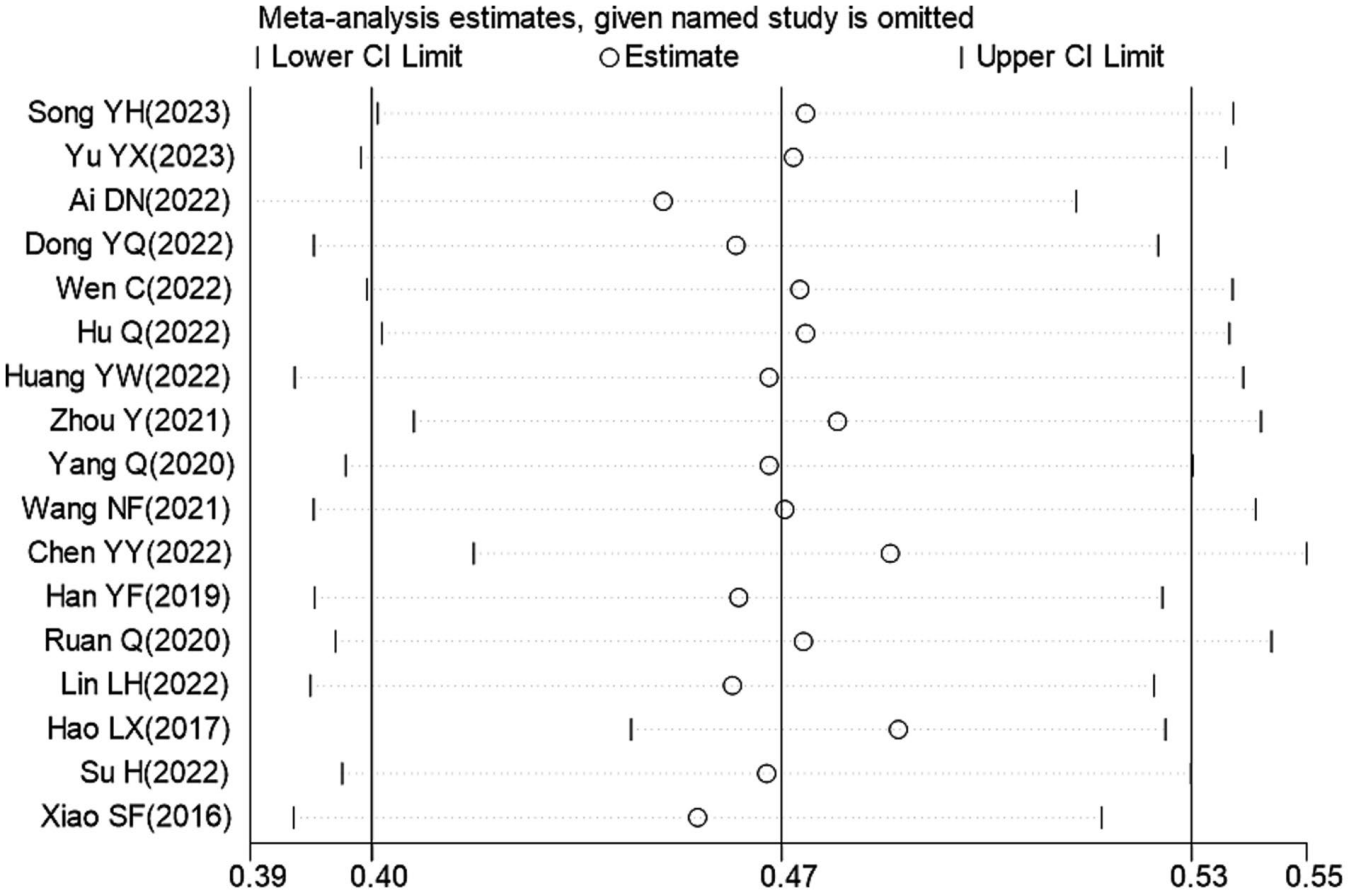
Sensitivity analysis of overall SCD prevalence in Chinese older adults.

We also conducted a sensitivity analysis by excluding one study at a time to assess whether the variation between studies affected the overall estimate. As depicted in Figure 3, none of the studies caused significant changes in the pooled prevalence. This indicates that the results of the Meta analysis are reliable and stable.

### 3.5 Publication bias

**Figure 4:**
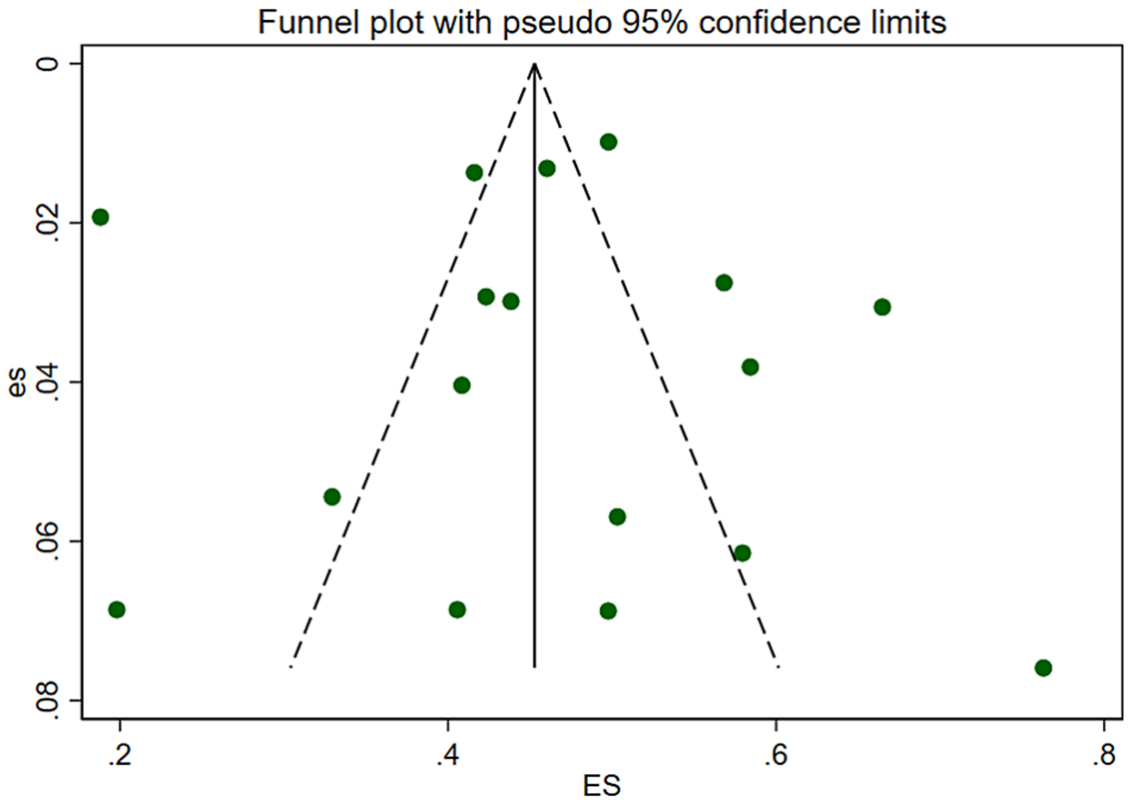
Funnel chart of overall SCD prevalence in Chinese older adults.

Visual inspection revealed slight signs of asymmetry in the funnel plot (Figure 4). Additionally, Egger’s test indicated no significant publication bias, with no statistical significance detected for t (*t* = 0.16) and (*p* = 0.878) values. Based on the visual evaluation and Egger’s test results, we concluded that the publication bias risk in this review was low.

## 4 Discussion

AD has become a public health priority in the context of aging (4, 5, 7). In recent years, SCD, a proposed preclinical stage of AD, has received considerable global interest (14, 16). To the best of our knowledge, this is the first systematic review to investigate SCD prevalence in older adults in China. We found that SCD prevalence was 46.4% among older Chinese adults, suggesting that this condition was particularly common within this population. Moreover, this result was higher than the SCD prevalence of 25.5 and 36.7% reported in the United States of America (48) and Australia (49), respectively, and comparable to that of 36.1–68.6% in Japan (50, 51). However, attention should be given to the fact that SCD prevalence in older adults may vary greatly between studies conducted in different countries due to the differences in sample source, sample size, geographical environment, and cultural background.

The 17 studies included in this review established strict inclusion and exclusion criteria when selecting study subjects to ensure homogeneity. All studies applied SCD diagnostic criteria consistent with those proposed by the Subjective Cognitive Decline Initiative (SCD-I) (13), ensuring the authenticity and accuracy of the reported SCD prevalence.

Additionally, our review found some distinctive findings during the subgroup analyzes. First, SCD prevalence was higher in women than in men. This difference may be because women are more sensitive to perceiving dynamic changes in their cognitive functioning than men; therefore, they are more likely to show concern about SCD (38, 40).

Second, SCD prevalence was positively correlated with increasing age, indicating that advanced age was associated with a higher risk of developing SCD. As individuals grow older, they may experience stressor overload, chronic inflammation (52, 53), and accelerated aging of brain cells (54), resulting in the gradual reduction of the brain’s cognitive reserve along with a continuous decline in cognitive function (55).

Third, older adults with an elementary school education or less had a higher SCD prevalence rate (62.8%). This observation suggests that higher education may be a protective factor for healthy cognitive functioning, consistent with previous findings (20, 34, 56, 57). This protective benefit could be attributed to the better knowledge base, stronger compensation function of the brain network, and increased neuronal vitality in older adults with higher education levels compared to those with an elementary school education or less (34, 35). These advantages could in turn lead to a better subjective cognitive status and relatively higher health literacy, making them more receptive to health knowledge and preventive and curative measures for SCD (34, 58). Therefore, medical professionals and policymakers should focus on the older population with lower education levels, develop a comprehensive cognitive screening and assessment process, and increase SCD awareness in the general population to improve health literacy and promote healthy behavior in older adults.

Fourth, this study found a higher prevalence of SCD in individuals with a BMI <18.5, at 59.3%. Although the difference in prevalence between BMI subgroups was only marginally statistically significant, these findings suggest that older adults with a lower BMI are more likely to experience SCD compared to those with a normal BMI. Previous research has also demonstrated a greater risk of concurrent malnutrition in older adults with a lower BMI (59), which can potentially contribute to persistent cognitive impairment (60). Therefore, it is recommended that the older population strive to maintain a normal BMI to preserve their ability to perform daily activities and retain good cognitive function.

Fifth, SCD prevalence in North and South China was >40.0%, implying a higher SCD prevalence in the Chinese population across different regions. Accordingly, physicians should conduct early comprehensive screening of subjective cognitive function in the Chinese population. This strategy will enable the timely detection and prevention of further cognitive function deterioration in individuals with SCD. Additionally, SCD prevalence in urban communities was found to be slightly lower than that in rural areas. This difference may be ascribed to the previously reported higher prevalence of cognitive disorders, including SCD, mild cognitive impairment, and dementia, in the rural areas of China (61).

Finally, manual laborers had a higher SCD prevalence than non-manual workers, possibly due to the better cognitive performance associated with higher education levels among the non-manual workers included in this study (34, 40, 41). Conversely, non-manual workers have been suggested to experience comparatively greater work stress, prolonged sedentary behavior, and a lack of appropriate physical activity (25, 62), potentially leading to excessive concern about their emotional state and cognitive functioning. Therefore, encouraging middle-aged and older populations to actively participate in community cultural activities is important (63). Such activities will help them avoid prolonged stress or negative emotions, ultimately enhancing their cognitive reserve as well as physical and mental well-being.

## 5 Research implications

Our systematic review and meta-analysis revealed that women, individuals of advanced age, those with low education levels, residents in the southern region and rural areas, and those with manual work before retirement may exhibit higher SCD prevalence. Consequently, studies focusing on these specific populations should be prioritized by future researchers. Furthermore, we observed that most original studies relied on the SCD questionnaire (including SCD-Q9, SCD-Q, and COWAT, etc.) alone to assess subjective cognitive function. However, using only one tool to evaluate SCD might not provide adequate diagnostic value (13, 16). Nonetheless, considering the wide and convenient clinical application of SCD-Q9/SCD-Q, subsequent studies should build upon this questionnaire as well as emphasize the development of an in-depth SCD assessment process and tools. Moreover, the aging and health trend will likely become exacerbated in China due to the increasing life expectancy accompanied by worsening health conditions (64). This trend will eventually impose substantial stress on the necessity for population-wide cognitive screening. Therefore, the widespread implementation of AI technology in SCD screening and diagnosis could be a potential research area to help address this issue.

## 6 Strength and limitations

This study has many strengths that are worth mentioning. To the best of our knowledge, this is the first systematic review and meta-analysis to evaluate SCD prevalence in older Chinese adults. Additionally, our review adopted a thorough search strategy encompassing nine databases in the Chinese and English languages. We also utilized a double review process, thereby enhancing the comprehensiveness of our search results on SCD epidemiology in China. Furthermore, the original studies included in our analysis were classified as moderate- to high-quality articles, ensuring the reliability of our findings. Finally, we conducted extensive subgroup analyzes to explore the epidemiological characteristics of SCD in older Chinese adults.

Nonetheless, our study has several limitations that should be addressed in future research. First, a substantial amount of heterogeneity was detected in the subgroup analyzes. However, this heterogeneity is unavoidable in the meta-analysis of epidemiological surveys (65). Second, the SCD assessment tools and diagnostic criteria employed in the included studies were not standardized, potentially introducing measurement bias and reducing our results’ reliability. Third, most studies we reviewed were conducted in eastern China, thus underrepresenting the country’s western region. Consequently, our sample does not adequately represent all 34 Chinese provinces, including provincial-level municipalities, autonomous regions, and special administrative regions. Lastly, although our findings are promising, they should be interpreted cautiously owing to the high heterogeneity observed among studies, moderate bias risk in certain studies, and the likelihood of mild publication bias.

## 7 Conclusion

Our review suggests that monitoring SCD prevalence in older adults in China is crucial for the effective planning and organization of related healthcare services. Based on the available literature, our systematic review and meta-analysis indicate that SCD has a prevalence of 46.4% in older Chinese adults. However, these values may be an underestimation of the true prevalence because of insufficient high-quality data. Therefore, future research should focus on investigating the incidence, risk factors, and causes of SCD. Furthermore, the relevant authorities in China are critically required to accelerate the development and implementation of a broad range of preventive and therapeutic strategies for SCD. Moreover, initiatives for the early screening, diagnosis, and prevention of SCD will further help alleviate the social care and economic burdens caused by AD in the older population in China.

## Data availability statement

The raw data supporting the conclusions of this article will be made available by the authors, without undue reservation.

## Author contributions

CX: Data curation, Investigation, Methodology, Software, Writing – original draft, Writing – review & editing. MH: Data curation, Investigation, Methodology, Software, Writing – original draft. LC: Investigation, Methodology, Software, Writing – original draft. ZC: Methodology, Software, Validation, Visualization, Writing – original draft. ZT: Methodology, Software, Validation, Visualization, Writing – original draft. HT: Methodology, Software, Validation, Visualization, Writing – original draft. QF: Conceptualization, Formal analysis, Supervision, Writing – review & editing. JL: Conceptualization, Formal analysis, Funding acquisition, Supervision, Visualization, Writing – review & editing.
